# Absolute cardiovascular risk in a Fiji medical zone

**DOI:** 10.1186/s12889-016-2806-6

**Published:** 2016-02-09

**Authors:** Rajat Gyaneshwar, Swaran Naidu, Magdalena Z. Raban, Sheetal Naidu, Christine Linhart, Stephen Morrell, Isimeli Tukana, Richard Taylor

**Affiliations:** 1 Viseisei Sai Health Centre, Viseisei village, Ba Province Fiji; 2 Fiji School of Medicine, College of Medicine, Nursing and Health Sciences, Fiji National University, Suva, Fiji; 3 Centre for Health Systems and Safety Research, Australian Institute of Health Innovation, Macquarie University, Sydney, NSW 2109 Australia; 4 School of Public Health and Community Medicine (SPHCM), Faculty of Medicine, University of New South Wales (UNSW), Sydney, NSW 2052 Australia; 5 Fiji Ministry of Health, Suva, Fiji

**Keywords:** Absolute cardiovascular risk, Non-communicable disease, Risk factors, Diabetes mellitus, Obesity, Fiji, Ethnic groups

## Abstract

**Background:**

The population of Fiji has experienced emergence of non-communicable disease (NCD) and a plateau in life expectancy over the past 20 years.

**Methods:**

A mini-STEPS survey (*n* = 2765) was conducted in Viseisei in Western Fiji to assess NCD risk factors (RFs) in i-Taukei (Melanesians) and those of Indian descent aged 25–64 years (response 73 %). Hypertension (HT) was defined as systolic blood pressure (BP) ≥140 mmHg or diastolic BP ≥90 mmHg or on medication for HT; type 2 diabetes mellitus (T2DM) as fasting plasma glucose ≥7.0 mmol/L or on medication for T2DM; and obesity as a body mass index (kilograms/height(metres)^2^) ≥30. Data were age-adjusted to 2007 Fiji Census. Associations between RFs and ethnicity/education were investigated. Comparisons with Fiji STEPS surveys were undertaken, and the absolute risk of a cardiovascular disease (CVD) event/death in 10 years was estimated from multiple RF charts.

**Results:**

NCD/RFs increased with age except excessive alcohol intake and daily smoking (women) which declined. Daily smoking was higher in men 33 % (95 % confidence interval: 31–36) than women 14 % (12–116); women were more obese 40 % (37–43) than men 23 % (20–26); HT was similar in men 37 % (34–40) and women 34 % (31–36), as was T2DM in men 15 % (13–17) and women 17 % (15–19). i-Taukei men had an odds ratio (OR) of 0.41 (0.28–0.58) for T2DM compared to Indians (1.00); and i-Taukei (both sexes) had a higher OR for obesity and low fruit/vegetable intake, daily smoking, excessive alcohol intake and HT in females. Increasing education correlated with lesser smoking, but with higher obesity and lower fruit/vegetable intake. Compared to the 2011 Fiji STEPS survey, no significant differences were evident in obesity, HT or T2DM prevalences. The proportion (40–64 years) classified at high or very high risk (≥20 %) of a CVD event/death (over 10 years) based on multiple RFs was 8.3 % for men (8.1 % i-Taukei, 8.5 % Indian), and 6.7 % for women (7.9 % i-Taukei, 6.0 % Indian).

**Conclusions:**

The results of the survey highlight the need for individual and community interventions to address the high levels of NCD/RFs. Evaluation of interventions is needed in order to inform NCD control policies in Fiji and other Pacific Island nations.

## Background

Non-communicable diseases (NCDs) are a major health problem in Fiji causing considerable premature mortality and limitation of improvement in life expectancy (LE) [1]. According to the World Health Organisation (WHO) NCDs include cardiovascular disease (CVD), diabetes, cancer and chronic lung disease, and are contributed to by risk factors (RFs) related to diet, exercise, and tobacco and alcohol consumption [2]. These RFs can be measured and monitored through population prevalence surveys, such as WHO STEPS surveys [3]. Control of NCD with lowering of population RFs and reduction in premature adult mortality can be accomplished through primary prevention by changing diet, exercise and consumption of tobacco and alcohol in populations using health promotion and policy regulation of marketing and supply. NCD control also involves the individual approaches of detection and treatment of cases of NCD (secondary and tertiary prevention) and management of those found to be at high risk of NCD, especially because of multiple RFs (primary prevention). The effectiveness of these interventions at a population level in the context of Fiji is yet to be established.

Fiji is a multi-ethnic society (Melanesian 57 %, Indian 38 %) of 827,900 people at the 2007 Census [4]. NCDs such as CVD and type 2 diabetes mellitus (T2DM) have been documented as public health concerns in Fiji since the 1970s [5–7]. In 2012 an NCD RF survey was undertaken in the Viseisei Health Zone, near Nadi in Fiji, to establish levels of NCDs, RFs (behavioural, physical and biochemical) and analyse predictors (age, sex, ethnicity and education) and absolute risk of a CVD event in this population. The purposes of the survey were to establish baseline means and prevalences of NCD and RFs, and absolute CVD risk, prior to a primary health care community intervention; and to identify those with NCD and at high risk for individual management. The survey was coordinated by the Viseisei Sai Health Centre which was developed as a charitable community health centre in collaboration with the Fiji Ministry of Health (MoH) and Fiji National University, College of Medicine, Nursing and Health Sciences. It serves the community health needs of a population of 7385, with a population ≥25 years of 3990 covering 14 census collection areas. The present study analyses the findings from this survey with respect to means and prevalences of NCD RFs and absolute risk of a CVD event, with differences by age, sex, and ethnicity, and comparisons with nationally representative data from Fiji STEPS surveys [8; Fiji MoH, Fiji NCD STEPS Survey 2002 unit record data supplied 2014, unpublished; Fiji MoH, Fiji NCD STEPS Survey 2011 unit record data supplied 2014, unpublished]. Apart from Papua New Guinea, Fiji has the largest population (837,000 at the 2007 Census) of Pacific Island States, where there is considerable similar morbidity and mortality from CVD, diabetes, cancer and chronic lung disease – especially in Polynesia and Micronesia.

## Methods

### Population and recruitment

The Viseisei Health Zone (VHZ) is near Nadi International Airport, in the Western Division of Fiji. The survey was carried out in 2012 to measure baseline NCD and RFs in the VHZ prior to the implementation of interventions targeting NCD and RFs in the community.

All VHZ community members aged 25–64 years were invited to participate if they were accessible by vehicle or a walk of <1 kilometre. The community was informed of the survey via community leaders, community health workers, and other key members of the community. 2675 individuals aged 25 to 64 years participated in the survey (72.7 % response rate). The sample characteristics are shown in Table 1. Ethics approval for the survey that included written consent was approved by the Fiji National Research Committee.

**Table 1:** Distribution (%) of characteristics of sampled adults aged 25 to 64 years Viseisei and Fiji

Characteristics	Viseisei, 2012	Fiji, 2011 STEPS	Fiji, 2002 STEPS
	(*n* = 2675)	(*n* = 2387)	(*n* = 4927)
	%	%	%
Response rate	72.7	53.3	96.3
Age (years)			
25–34	27.5	24.8	28.8
35–44	25.2	27.8	31.0
45–54	27.3	30.2	23.4
55–64	20.1	17.2	16.8
Sex			
Male	43.9	44.0	41.0
Female	56.2	56.0	59.0
Ethnicity			
I-Taukei	41.0	56.1	57.2
Indo-Fijian	58.4	43.9	42.8
Highest level of education			
Primary or less	30.6	–	47.8
Any high school	35.0	–	44.3
Vocational training	24.1	–	4.7
University	10.3	–	3.2

Fiji NCD STEPS Survey 2002 [8; Fiji MoH, Fiji NCD STEPS Survey 2002 unit record data supplied 2014, unpublished] and 2011 [Fiji MoH, Fiji NCD STEPS Survey 2011 unit record data supplied 2014, unpublished]

### Measurements

Participants were recruited to the study on the day preceding the survey and advised to fast overnight. On the day of the survey trained staff administered the behavioural questionnaire and took physical and biochemical measurements.

The questionnaire documented tobacco use: current and daily smoking; alcohol consumption: in the last 12 months, and ≥4 drinks for women or ≥5 for men at ≥1 drinking session in the previous month; and intake of fruit and vegetables (<5 servings/day), using a modified version of the WHO STEPS instrument [3]. Height, weight, and waist and hip circumferences were measured. Body mass index (BMI) was defined as weight (kilograms) divided by height (meters) squared (kg/m^2^). Participants with a BMI ≥30 were classified as obese [3]. The WHO cut-offs, indicating a substantially increased risk of metabolic complications [9], were applied as measures of central adiposity: waist circumference (men 102 cm; women 88 cm) and waist-to-hip ratio (men 0.9 cm; women 0.85 cm).

Three blood pressure (BP) readings were taken for each respondent. Mean systolic blood pressure (SBP) and diastolic blood pressure (DBP) were calculated from the first two readings if the difference between them was <10 mmHg, and from the second two readings of the difference between the first and second reading was ≥10 mmHg. Hypertension (HT) was defined as a SBP ≥140 mmHg or DBP ≥90 mmHg or a respondent currently taking medication for HT [3].

Fasting plasma glucose (FPG) was estimated using Optium Xceed (Abbott) blood glucose system which is calibrated to provide plasma glucose equivalents from whole blood (either capillary or venous), and T2DM was defined as FPG ≥7.0 mmol/L or taking medication for T2DM [3]. This definition is the same as used for the WHO Fiji 2011 STEPS survey. High total cholesterol was defined as a total cholesterol ≥5.0 mmol/L or taking medication for high cholesterol [3].

Ethnicity was defined as: i-Taukei (formerly indigenous Fijians), or Indo-Fijian (Fijians of Indian descent). For the present study only subjects aged 25–64 years and of i-Taukei or Indo-Fijian ethnicity are included in the analysis. The main two ethnicities comprise 57 and 38 % respectively of the Fiji 2007 Census population [4]. The ‘Other’ ethnicity was not included as it is heterogeneous consisting of other Pacific Islanders, Europeans and Asians, and comprises only 5 % of the total Fiji population (2007 Census) [4]. Education was defined as highest level of education achieved: primary school or lower, any high school, vocational training, or university.

### Data analysis

Means and prevalences were calculated for continuous and categorical variables, respectively, with 95 % confidence intervals. Of the 2675 participants, 2525 had height, weight, waist and hip circumference measurements, 2549 had BP measurements and 2505 had FPG and cholesterol measurements. Prevalences of NCD and RFs for VHZ were estimated by age group for both sexes. A test for linear trend of age group for each NCD and RF was conducted using age group as an ordinal independent variable in linear or logistic regression for means and prevalences, respectively. A statistically significant beta estimate from the regression model for age group indicates a significant age trend.

Estimates of NCD and RFs for the 2012 VHZ population were weighted using the age, sex and ethnicity distribution of the 2007 Fiji Census population [Fiji Islands Bureau of Statistics. Fiji Census 2007: Population table by sex, age, ethnicity, urban/rural: supplied 2014, unpublished]. These estimates were compared to those of the 2011 Fiji STEPS survey weighted to the age, sex, ethnicity and rural/urban distribution of the 2007 Fiji Census population. The Fiji 2002 STEPS survey was weighted to the age, sex, ethnicity and rural/urban distributions of the 1996 Fiji Census population. Weighting of STEPS surveys to the most recent previous census population structure improves representativeness.

For the Fiji 2002 STEPS, T2DM was defined as fasting blood glucose of ≥6.1 mmol/L, since glucose was measured from a whole blood sample using a system that did not provide a plasma glucose equivalent. The mean fasting blood glucose level was adjusted by a factor of 1.11 to give a plasma level approximation, as recommended by the International Federation of Clinical Chemistry (IFCC) [10].

Ethnic and education as determinants of NCD and RFs in VHZ were investigated by comparing differences and trends in RFs across categories. Ethnic comparisons (i-Taukei, Indo-Fijians) are expressed as odds ratios (OR) from logistic regression (adjusted for age as a continuous variable). Analysis of NCD and risk factors and education attainment considered sex as an effect modifier which was accounted for by stratification. Age group and ethnicity were considered as (potential) confounders and controlled for in logistic regressions with the NCD or risk factor (RF) as the dichotomous (y) dependent (outcome) variable, and the confounders (age, ethnicity), and study factor (education), as categorical independent (x) variables, except that education was also used as an ordinal variable to determine linear trend.

The absolute CVD risk at the population level was estimated by applying the WHO/International Society of Hypertension (WHO/ISH) Western Pacific Region Group B risk prediction charts to respondents aged 40 years and over [11, 12]. The WHO/ISH risk prediction charts give a percentage risk of a CVD event in 10 years based on an individual’s age, sex, smoking status, T2DM status, SBP and total cholesterol levels [11, 12]. Individuals were categorised as low (<10 %), medium (10 % to <20 %), high (20 % to <30 %), and very high (≥30 %) risk of a CVD event.

Data were analysed using Statistical Analysis System (SAS) version 9.4 [13] and Microsoft Excel [14] software.

## Results

For men, mean waist circumference and waist:hip ratio, but not BMI, increased with age, as did mean BP and HT prevalence, mean FPG and T2DM prevalence and blood cholesterol (mean and prevalence of elevated levels); excessive alcohol intake declined with age (Table 2). For women, all NCD RFs increased with age, except daily smoking and excessive alcohol intake, which declined with age (Table 3). Daily smoking, excessive alcohol use and low fruit and vegetable intake were more prevalent among men than women. Women had higher means and prevalences of measures of obesity than men (BMI 28.9 vs 26.4; obesity 39.9 % vs 23.2 %), but there were no sex differences in prevalences of HT (32–33 %) or T2DM (15–17 %) (Tables 2 and 3).

**Table 2:** Males: non-communicable disease and risk factors by age group, 25 to 64 years in Viseisei and Fiji

NCD and risk factors	Means and prevalences with 95 % confidence intervals							
	Age specific data and trend by age group					Age std to: 2007 census		1996 census
	Viseisei 2012 survey						Fiji 2011^*^	Fiji 2002^*^
	25–34 yrs	35–44 yrs	45–54 yrs	55–64 yrs	Age: linear trend^‡‡^	25–64 std^#^	25–64 std^‡^	25–64 std^†^
	(*n* = 319)	(*n* = 298)	(*n* = 316)	(*n* = 240)		(*n* = 1173)	(*n* = 1050)	(*n* = 2019 )
Daily smoker %	34.2	33.6	31.0	29.2	0.92 (*p* = 0.16)	33.4	26.2	28.8
	29.0–39.4	28.2–38.9	25.9–36.1	23.4–34.9		30.5–36.3	23.3–29.2	26.7–30.9
Excessive alcohol %	55.8	46.9	33.7	22.0	**0.61** (*p* < 0.0001)	43.7	19.9	–
	49.9–61.6	41.1–52.6	28.4–39.0	16.7–27.3		40.5–46.8	17.1–22.7	
Low fruit & vegetable %	70.9	66.4	74.0	70.5	1.04 (*p* = 0.56)	72.4	47.8	–
	65.9–75.9	61.0–71.9	69.1–78.8	64.7–76.3		69.7–75.1	44.2–51.3	
Mean BMI kg/m^2^	25.0	26.4	26.5	25.6	0.23 (*p* = 0.11)	26.4	26.4	25.1
	24.4–25.6	25.8–27.0	25.8–27.1	25.0–26.3		26.1–26.8	26.0–26.8	24.9–25.3
BMI ≥ 25 kg/m^2^ %	40.1	54.4	52.8	50.0	1.10 (*p* = 0.10)	56.7	58.7	46.3
	34.7–45.5	48.7–60.0	47.3–58.4	43.7–56.3		53.6–59.8	55.6–61.9	44.0–48.6
BMI ≥ 30 kg/m^2^ %	15.9	19.9	25.4	19.0	1.12 (*p* = 0.11)	23.2	22.0	12.9
	11.7–20.1	15.1–24.6	20.5–30.4	14.0–24.1		20.4–25.9	19.4–24.6	11.4–14.4
Mean waist cm	88.0	92.2	94.7	94.4	**2.28** (*p* < 0.0001)	92.6	91.2	86.0
	86.6–89.3	90.8–93.6	93.2–96.2	92.9–95.9		91.8–93.3	88.4–94.1	85.5–86.6
High waist %^║^	10.7	18.4	29.1	23.8	**1.40** (*p* < 0.0001)	21.9	18.4	9.3
	7.1–14.2	13.8–23.0	23.9–34.2	18.3–29.3		19.3–24.6	16.0–20.7	8.0–10.6
Mean waist:hip	0.87	0.90	0.91	0.93	**0.02** (*p* < 0.0001)	0.89	0.93	0.90
	0.86–0.88	0.89–0.90	0.91–0.92	0.92–0.93		0.89–0.90	0.90–0.96	0.90–0.91
High^§^ waist:hip%	28.3	46.0	64.9	70.1	**1.88** (*p* < 0.0001)	47.0	62.0	50.1
	23.1–33.5	40.0–51.9	59.5–70.3	64.2–76.0		43.8–50.1	58.9–65.1	47.8–52.5
Mean SBP mmHg	125	129	135	143	**5.91** (*p* < 0.0001)	131.4	132	127
	123–126	127–130	133–137	139–146		130–132	131–133	127–128
Mean DBP mmHg	79.2	85.2	87.3	87.0	**2.67** (*p* < 0.0001)	83.9	81.0	73.5
	77.9–80.5	83.9–86.4	86.0–88.6	85.4–88.6		81.5–82.9	80.2–81.7	72.9–74.0
HT (%)^¶^	23.2	34.9	47.3	55.4	**1.61** (*p* < 0.0001)	37.2	36.6	19.1
	18.3–28.1	29.3–40.6	41.7–53.0	49.0–61.8		34.2–40.3	33.3–40.0	17.4–20.9
Mean FBG mmol/L	5.3	5.6	6.5	7.0	**0.60** (*p* < 0.0001)	5.8	6.4	6.2
	5.1–5.5	5.4–5.8	6.2–6.8	6.6–7.3		5.7–5.9	6.1–6.8	6.1–6.4
Diabetes % ^**^	6.3	11.6	27.6	35.6	**2.05** (*p* < 0.0001)	15.1	13.8	16.3
	3.5–9.1	7.7–15.4	22.4–32.7	29.5–41.8		13.0–17.2	11.4–16.2	13.8–18.9
Mean chol mmol/L	4.6	5.2	5.3	5.3	**0.22** (*p* < 0.0001)	5.0	–	5.4
	4.5–4.8	5.0–5.3	5.2–5.5	5.1–5.5		4.9–5.1		5.3–5.5
High chol % ^††^	9.5	18.4	24.5	22.1	**1.37** (*p* < 0.0001)	17.5	–	–
	6.1–12.9	13.7–23.0	19.5–29.4	16.7–27.4		15.1–19.8		

Fiji data from WHO STEPS surveys 2002 [Fiji MoH, Fiji NCD STEPS Survey 2002 unit record data supplied 2014, unpublished] and 2011 [Fiji MoH, Fiji NCD STEPS Survey 2011 unit record data supplied 2014, unpublished]. Prevalence or mean with 95 % confidence intervals. Statistically significant linear trend *p* < 0.05 are in bold

*BMI* body mass index, *BP* blood pressure, *SBP* systolic BP, *DBP* diastolic BP, *HT* hypertension, *FBG* fasting plasma glucose, *chol* total plasma cholesterol

‡‡Linear trend represents average change in odds ratio between age groups for prevalence estimates and average change in measurement between age groups for mean estimates. ║Waist circumference >102 cm for males. § Waist to hip ratio ≥0.9 for males

Std *Standardised for age and ethnicity to Fiji 2007 census population [4, Fiji Islands Bureau of Statistics. Fiji Census 2007: Population table by sex, age, ethnicity, urban/rural: supplied 2014, unpublished]; ‡Standardised for age, ethnicity and rural/urban locality to Fiji 2007 census population. †Standardised for age, ethnicity and rural/urban locality to Fiji 1996 census population [15]; ¶ Systolic BP ≥ 140 mmHg and/or diastolic BP ≥ 90 mmHg and/or on medication for HT

** Viseisei 2012 and Fiji STEPS 2011 [Fiji MoH, Fiji NCD STEPS Survey 2011 unit record data supplied 2014, unpublished]: FPG ≥7.0 mmol/L and/or on medication for diabetes; Fiji STEPS 2002 [Fiji MoH, Fiji NCD STEPS Survey 2002 unit record data supplied 2014, unpublished]: FBG ≥6.1 mmol/L and/or on medication for diabetes. ††Total cholesterol level ≥5.0 mmol/L and/or on medication for high cholesterol

**Table 3:** Females: non-communicable disease and risk factors by age group, 25 to 64 years in Viseisei and Fiji

NCD and risk factors	Means and prevalences with 95 % confidence intervals							
	Age specific data and trend by age group					Age std to: 2007 census		1996 census
	Viseisei 2012 survey						Fiji 2011^*^	Fiji 2002^*^
	25–34 yrs	35–44 yrs	45–54 yrs	55–64 yrs	Age: linear trend^‡‡^	25–64 std^#^	25–64 std^‡^	25–64 std^†^
	(*n* = 319)	(*n* = 298)	(*n* = 316)	(*n* = 240)		(*n* = 1173)	(*n* = 1050)	(*n* = 2019)
Daily smoker %	7.7	10.4	3.9	4.0	**0.75** (*p* = 0.004)	8.8	5.9	4.5
	5.1–10.2	7.3–13.5	2.0–5.7	1.8–6.3		7.1–10.5	4.3–7.4	3.8–5.2
Excessive alcohol %	10.4	7.9	1.2	1.3	**0.45** (*p* < 0.0001)	7.6	2.8	–
	7.4–13.3	5.1–10.6	0.2–2.3	0.0–2.7		6.0–9.2	1.8–3.8	
Low fruit & vegetable %	63.1	66.6	66.5	74.2	**1.16** (*p* = 0.005)	68.7	43.6	–
	58.5–67.7	61.8–71.4	61.9–71.1	69.2–79.2		66.3–71.2	40.5–46.6	
Mean BMI kg/m^2^	27.0	29.1	28.6	29.0	**0.56** (*p* = 0.0005)	28.9	29.3	28.0
	26.3–27.7	28.5–29.8	27.9–29.3	28.3–29.6		28.6–29.3	28.9–29.7	27.8–28.3
BMI ≥ 25 kg/m^2^ %	52.0	69.6	62.7	71.0	**1.20** (*p* = 0.0006)	69.2	74.8	68.0
	47.2–56.8	64.9–74.3	58.0–67.4	65.9–76.2		66.7–71.6	72.4–77.3	66.2–69.8
BMI ≥ 30 kg/m^2^ %	28.1	41.4	37.1	39.7	**1.15** (*p* = 0.006)	39.9	41.7	33.5
	23.6–32.6	36.3–46.5	32.4–41.8	34.0–45.3		37.1–42.6	39.0–44.4	31.8–35.3
Mean waist cm	87.1	92.2	92.2	94.7	**2.31** (*p* < 0.000)	92.5	92.8	87.1
	85.6–88.6	90.8–93.7	90.8–93.5	93.2–96.2		91.6–93.3	91.8–93.8	86.6–87.6
High waist %^║^	46.4	63.0	60.1	68.0	**1.30** (*p* < 0.0001)	61.9	63.6	47.3
	41.4–51.3	58.0–68.0	55.4–64.9	62.7–73.4		59.3–64.6	60.7–66.5	45.4–49.2
Mean waist:hip	0.84	0.86	0.87	0.89	**0.02** (*p* < 0.0001)	0.86	0.89	0.86
	0.83–0.85	0.85–0.86	0.86–0.88	0.88–0.90		0.86–0.87	0.88–0.89)	0.86–0.87
High^§^ waist:hip %	46.6	54.2	64.4	72.9	**1.46** (*p* < 0.0001)	58.8	72.9	54.8
	41.6–51.6	49.0–59.4	59.7–69.0	67.7–78.0		56.1–61.5	70.3–75.6	52.9–56.7
Mean SBP mmHg	117	126	139	152	**11.6** (*p* < 0.0001)	130.2	129	125
	116–118	124–128	136–141	149–155		129–132	128–131	124–126
Mean DBP mmHg	76.3	81.5	85.2	89.9	**4.46** (*p* < 0.0001)	82.2	80.5	74.4
	75.3–77.3	80.3–82.8	83.9–86.5	88.4–91.5		81.5–82.9	79.8–81.3	73.9–74.9
HT (%)^¶^	10.0	27.1	47.9	74.3	**2.88** (*p* < 0.0001)	33.7	33.3	20.0
	7.0–12.9	22.5–31.7	43.0–52.8	69.3–79.3		31.2–36.2	30.4–36.1	18.6–21.5
Mean FBG mmol/L	5.4	6.1	7.2	6.9	**0.59** (*p* < 0.0001)	6.2	6.4	6.4
	5.3–5.6	5.8–6.3	6.9–7.6	6.6–7.2		6.1–6.3	6.2–6.6	6.2–6.2
Diabetes % ^**^	5.3	14.5	30.5	33.7	**1.98** (*p* < 0.0001)	17.3	16.7	16.6
	3.1–7.6	10.8–18.2	25.9–35.0	28.2–39.1		15.3–19.3	14.5–18.9	14.6–18.6
Mean chol mmol/L	4.3	4.7	5.2	5.5	**0.42** (*p* < 0.0001)	4.8	–	5.1
	4.2–4.5	4.5–4.8	5.1–5.3	5.4–5.7		4.7–4.9		5.0–5.1
High chol % ^††^	5.3	11.4	21.2	29.7	**1.92** (*p* < 0.0001)	14.7	–	–
	3.1–7.6	8.0–14.7	17.2–25.2	24.4–34.9		12.8–16.6		

Fiji data from WHO STEPS surveys 2002 [Fiji MoH, Fiji NCD STEPS Survey 2002 unit record data supplied 2014, unpublished] and 2011 [Fiji MoH, Fiji NCD STEPS Survey 2011 unit record data supplied 2014, unpublished]. Prevalence or mean with 95 % confidence intervals. Statistically significant linear trend *p* < 0.05 are in bold

*BMI* body mass index, *BP* blood pressure, *SBP* systolic BP, *DBP* diastolic BP, *HT* hypertension, *FBG* fasting plasma glucose, *chol* total plasma cholesterol

‡‡Linear trend represents average change in odds ratio between age groups for prevalence estimates and average change in measurement between age groups for mean estimates. ║Waist circumference >88 cm for females. § Waist to hip ratio ≥0.85 for females

Std *Standardised for age and ethnicity to Fiji 2007 census population [4]; ‡Standardised for age, ethnicity and rural/urban locality to Fiji 2007 census population [4, Fiji Islands Bureau of Statistics. Fiji Census 2007: Population table by sex, age, ethnicity, urban/rural: supplied 2014, unpublished]. †Standardised for age, ethnicity and rural/urban locality to Fiji 1996 census population [15]; ¶ Systolic BP ≥ 140 mmHg and/or diastolic BP ≥ 90 mmHg and/or on medication for HT

**Viseisei 2012 and Fiji STEPS 2011 [Fiji MoH, Fiji NCD STEPS Survey 2011 unit record data supplied 2014, unpublished]: FPG ≥7.0 mmol/L and/or on medication for diabetes; Fiji STEPS 2002 [Fiji MoH, Fiji NCD STEPS Survey 2002 unit record data supplied 2014, unpublished]: FBG ≥6.1 mmol/L and/or on medication for diabetes. ††Total cholesterol level ≥5.0 mmol/L and/or on medication for high cholesterol

Compared to the 2011 Fiji STEPS survey, men in the VHZ survey had higher rates of daily smoking, excessive alcohol intake, and lower fruit and vegetable intake, while the means and prevalences of other RFs were similar (Table 2). Women in the VHZ survey had a higher prevalence of excessive alcohol intake and lower fruit and vegetable consumption, but lower prevalence of high waist-to-hip ratio (Table 3). There were no significant differences in obesity (based on BMI), HT or T2DM prevalences. Comparison of both the VHZ 2012 and Fiji 2011 STEPS surveys with the Fiji STEPS 2002 indicate an increase in obesity and HT in both sexes over the decade.

Based on age-adjusted ORs, i-Taukei men were significantly more likely than Indo-Fijian men to have low fruit and vegetable intake, obesity, or high waist circumference, but less likely to have T2DM (Table 4). For women, i-Taukei had higher ORs compared to Indo-Fijians for daily smoking, excessive alcohol intake, low fruit and vegetable intake, obesity, high waist circumference and waist-hip ratio, and HT prevalence; but the OR for prevalence of T2DM and high cholesterol were not significantly different (Table 4). Adjusted for age and ethnicity, increasing education in men showed a statistically significant trend for lower daily smoking, but a significant positive trend for obesity, high waist circumference, low fruit and vegetable intake, and HT. For women, increasing education showed significant negative linear trend for high waist-to-hip ratio and low fruit and vegetable intake, but a positive trend for excessive alcohol intake (Table 4).

**Table 4:** Association of non-communicable disease risk factors with ethnicity and education, Viseisei Health Zone, Fiji, 2012

NCD and Risk factors by sex	Odds ratio (95 % CI)						
	Ethnicity (age adj)		Education (age and ethnicity adjusted)				
	I-Taukei	Indo-Fijian	Primary or less	Any high school	Vocational training	University	Test for trend^+^
Males							
Daily smoking	1.19 (0.93–1.53)	1.00	**3.09** (1.83–5.22)	**2.45** (1.50–4.00)	**1.91** (1.14–3.20)	1.00	**0.73** (p < 0.0001)
Excessive alcohol	1.13 (0.87–1.45)	1.00	1.50 (0.93–2.44)	**1.90** (1.22–2.97)	1.44 (0.90–2.30)	1.00	0.89 (*p* = 0.10)
Low fruit & vegetable	**2.39** (1.81–3.16)	1.00	**2.29** (1.41–3.73)	1.43 (0.92–2.22)	**1.69** (1.06–2.69)	1.00	**0.81** (*p* = 0.005)
Obesity BMI ≥ 30 kg/m^2^	**6.88** (4.85–9.76)	1.00	**0.35** (0.19–0.64)	**0.41** (0.24–0.70)	0.67 (0.39–1.16)	1.00	**1.45** (*p* < 0.0001)
High waist* circumference	**5.09** (3.64–7.11)	1.00	**0.40** (0.21–0.75)	0.62 (0.35–1.10)	0.90 (0.50–1.61)	1.00	**1.40** (*p* = 0.0004)
High waist-hip ratio†	0.89 (0.68–1.15)	1.00	0.65 (0.40–1.07)	0.89 (0.56–1.41)	0.87 (0.54–1.41)	1.00	1.14 (*p* = 0.08)
HT‡	1.10 (0.85–1.42)	1.00	0.64 (0.39–1.05)	0.63 (0.40–1.01)	0.89 (0.55–1.44)	1.00	**1.18** (*p* = 0.03)
Diabetes║	**0.41** (0.28–0.58)	1.00	1.21 (0.53–2.76)	1.54 (0.69–3.43)	2.11 (0.93–4.78)	1.00	1.12 (*p* = 0.26)
High cholesterol§	1.08 (0.79–1.49)	1.00	0.70 (0.38–1.31)	0.71 (0.40–1.28)	1.00 (0.55–1.81)	1.00	1.16 (*p* = 0.11)
Females							
Daily smoking	**51.2** (16.1–162.5)	1.00	**6.04** (2.02–18.09)	**3.56** (1.33–9.54)	**6.35** (2.39–16.84)	1.00	0.80 (*p* = 0.09)
Excessive alcohol	**22.02** (8.82–54.96)	1.00	0.83 (0.34–2.03)	**0.34** (0.17–0.70)	0.93 (0.50–1.75)	1.00	**1.36** (*p* = 0.03)
Low fruit & vegetable	**2.34** (1.84–2.97)	1.00	**1.86** (1.16–2.98)	1.22 (0.81–1.84)	1.49 (0.81–1.84)	1.00	**0.88** (*p* = 0.06)
Obesity BMI ≥ 30 kg/m^2^	**4.65** (3.67–5.89)	1.00	1.07 (0.65–1.76)	1.15 (0.74–1.77)	1.34 (0.86–2.09)	1.00	1.03 (*p* = 0.65)
High waist * circumference	**5.10** (3.97–6.56)	1.00	1.58 (0.97–2.59)	**1.60** (1.03–2.48)	1.47 (0.94–2.30)	1.00	0.90 (*p* = 0.13)
High waist-hip ratio†	**1.97** (1.57–2.47)	1.00	**1.77** (1.11–2.82)	**1.78** (1.18–2.69)	1.32 (0.87–2.00)	1.00	**0.83** (*p* = 0.006)
HT‡	**1.51** (1.17–1.94)	1.00	1.20 (0.68–2.13)	1.22 (0.72–2.08)	1.08 (0.63–1.86)	1.00	0.94 (*p* = 0.43)
Diabetes║	0.77 (0.58–1.03)	1.00	1.26 (0.62–2.54)	1.33 (0.69–2.59)	1.28 (0.65–2.53)	1.00	0.97 (*p* = 0.73)
High cholesterol§	1.33 (0.99–1.80)	1.00	0.94 (0.47–1.88)	0.84 (0.44–1.61)	0.98 (0.51–1.89)	1.00	1.03 (*p* = 0.80)

Statistically significant *p* < 0.05 Odds Ratios are in bold. OR for trend <1.00 indicates decreasing association with increasing education, and OR > 1.00 indicates increasing association with increasing education. + Linear trend as average increase or decrease in weighted OR across ordered categories

*BMI* body mass index, *SBP* systolic blood pressure, *DBP* diastolic blood pressure, *HT* hypertension

*Waist circumference >102 cm for males and >88 cm for females. †Waist to hip ratio ≥0.9 for males and ≥0.85 for females

‡ Systolic BP ≥ 140 mmHg and/or diastolic BP ≥ 90 mmHg and/or on medication for HT

║FPG ≥ 7.0 mmol/L and/or on medication for diabetes. §Total cholesterol level ≥5.0 mmol/L and/or on medication for cholesterol

Absolute CVD risk categories in those aged over 40 years were calculated from risk charts based on age, sex, BP, T2DM, smoking and cholesterol categories. For men, 5.3 % (95%CI: 3.6–6.9) were classified at very high risk (≥30 %) and 3.0 % (95%CI: 1.7–4.2) at high risk (≥20– < 30 %) of a CVD event (total 8.3 % at high or very high risk); for women 4.0 % (95 % CI: 2.7–5.2) were classified at very high risk, and 2.7 % (95 % CI: 1.7–3.7) at high risk of a CVD event (total at 6.7 % at high or very high risk) (Table 5). There were no significant ethnic differences in those classified at high or very high risk of a CVD event.

**Table 5:** Proportions of the population in various cardiovascular risk categories by age, sex and ethnicity, Viseisei Health Zone, Fiji, 2012

Sex/Age (years)/Ethnicity	Cardiovascular event* risk category			
	Very high risk: ≥30 %	High risk: 20 % to <30 %	Medium risk: 10 to <20 %	Low risk: <10 %
	% (95 % CI)	% (95 % CI)	% (95 % CI)	% (95 % CI)
Males				
40–49 (*n* = 282)	1.1 (0–2.3)	0.7 (0–1.7)	12.1 (8.3–15.9)	86.2 (82.1–90.2)
50–59 (*n* = 271)	4.4 (2.0–6.9)	5.5 (2.8–8.3)	18.8 (14.2–23.5)	71.2 (65.8–76.6)
60–64 (*n* = 99)	25.3 (16.7–33.8)	5.1 (0.7–9.4)	24.2 (15.8–32.7)	45.5 (35.6–55.3)
40–64† (*n* = 652)	5.3 (3.6–6.9)	3.0 (1.7–4.2)	16.3 (13.5–19.2)	75.4 (72.1–78.7)
I-Taukei† (*n* = 259)	4.3 (2.0–6.7)	3.8 (1.6–6.0)	13.1 (9.0–17.2)	78.7 (73.8–83.6)
Indo-Fijian† (*n* = 385)	6.0 (3.8–8.2)	2.5 (1.0–3.9)	18.2 (14.3–22.1)	73.3 (68.9–77.8)
Females				
40–49 (*n* = 405)	1.7 (0.5–3.0)	0.5 (0–1.2)	1.7 (0.5–3.0)	96.0 (94.2–97.9)
50–59 (*n* = 357)	4.8 (2.6–7.0)	2.5 (0.9–4.1)	6.2 (3.7–8.7)	86.6 (83.0–90.1)
60–64 (*n* = 120)	12.5 (6.6–18.4)	14.2 (7.9–20.4)	8.3 (3.9–13.3)	65.0 (56.5–73.5)
40–64† (*n* = 882)	4.0 (2.7–5.2)	2.7 (1.7–3.7)	4.0 (2.8–5.3)	89.3 (87.4–91.3)
I-Taukei† (*n* = 315)	4.2 (2.0–6.3)	3.7 (1.8–5.6)	4.3 (2.2–6.4)	87.9 (84.4–91.3)
Indo-Fijian† (*n* = 563)	3.9 (2.4–5.4)	2.1 (1.0–3.2)	3.9 (2.4–5.5)	90.1 (87.7–92.4)

*Risk of cardiovascular event or death risk over 10 years based on

WHO/International Society of Hypertension Western Pacific Region Group B risk prediction charts

WHO/ISH Risk Prediction Charts for 14 WHO Epidemiological Sub-Regions. WHO 2007 [11]

† Age adjusted to total Fiji population at the 2007 Census [4]

## Discussion

Prevalence of RFs in the VHZ in 2012 varied by age, sex, ethnicity and education level. Most RF and NCD prevalences increased with age, except excessive alcohol intake and daily smoking (women) which declined with increasing age. Daily smoking prevalence was higher in men than women, but women were more obese than men. The prevalences of HT and T2DM were similar in both sexes. i-Taukei men had less than half the prevalence of T2DM than Fijians of Indian descent; and i-Taukei had higher ORs for indices of obesity, low fruit/vegetable intake (both sexes), and daily smoking, excessive alcohol intake and HT in females (age-adjusted). Increasing education correlated with lower smoking rates, but higher ORs for obesity, high waist circumference, waist-hip ratio and lower fruit and vegetable intake (adjusted for age and ethnicity). Compared to the Fiji 2011 STEPS survey, alcohol intake, low fruit/vegetable intake and daily smoking (men) were higher in VHZ than nationally, but no significant differences in obesity (based on BMI), HT or T2DM prevalences (adjusted for age, ethnicity, urban/rural). The proportion (40–64 years) classified at high risk or very high risk (≥20 %) of CVD event (over 10 years) based on multiple RF estimation was 8.3 % for men and 6.7 % for women.

Despite lower obesity using the standard WHO definition, Fijians of Indian descent had higher prevalence of diabetes mellitus than i-Taukei, associated with the well described propensit y of south Asians to develop diabetes at lower BMI than several other ethnicities [15]. Compared to Fijians of Indian descent, i-Taukei had lower fruit and vegetable intake, and higher tobacco and alcohol consumption. These differences require further investigation and indicate that targeted heath education is required. Differences by education suggest that health promotion messages concerning tobacco smoking are having an effect, but, so far, not those related to diet.

To simplify analyses and improve comparability, the age groups were restricted to 25–64 years, and the ‘Other’ ethnic group was excluded from analyses. The ‘Other’ ethnicity is heterogeneous consisting of other Pacific Islanders, Europeans and Asians, and comprises only 5 % of the total Fiji population (2007 Census) [4].

In order to improve national representativeness the Fiji STEPS surveys were adjusted (using case weights) to the closest previous Fiji census which were used for the sampling frame, since the actual age, sex, ethnic and rural/urban structure of the survey respondents differed to some extent. Thus the 2007 Fiji Census [Fiji Islands Bureau of Statistics. Fiji Census 2007: Population table by sex, age, ethnicity, urban/rural: supplied 2014, unpublished] was used for the WHO Fiji 2011 STEPS survey and the 1996 Fiji Census [16] was used for the WHO Fiji 2002 STEPS survey [8, Fiji MoH, Fiji NCD STEPS Survey 2002 unit record data supplied 2014, unpublished].

The VHZ 2012 survey is adjusted for age and ethnicity by sex to the 2007 Fiji Census [Fiji Islands Bureau of Statistics. Fiji Census 2007: Population table by sex, age, ethnicity, urban/rural: supplied 2014, unpublished] in order to minimise confounding when comparing with the WHO Fiji 2011 STEPS survey [8, Fiji MoH, NCD STEPS Survey 2011 tabulations supplied 2014, unpublished] for these variables.

Compared to Fiji 2011 STEPS survey, alcohol intake, low fruit/vegetable intake and daily smoking (men) were higher in VHZ than nationally, but there were no significant differences in obesity (based on BMI), HT or T2DM prevalences - adjusted for age, ethnicity, urban/rural to the Fiji 2007 Census. Comparison of both the VHZ 2012 and Fiji 2011 STEPS surveys with the Fiji STEPS 2002 suggest an increase in obesity and HT in both sexes over the decade, although the 2002 STEPS is adjusted to the previous Fiji Census (1996) and some of this increase may be related to demographic changes.

Relatively high prevalences of NCD and RFs in Fiji were documented by population NCD and RF surveys from the 1960s [17] and 1980s [18], as well as the WHO NCD Fiji STEPs surveys 2002 and 2011 [8, Fiji MoH, Fiji NCD STEPS Survey 2011 unit record data supplied 2014, unpublished]. Studies of trends over 30 years (to 2011) in diabetes and obesity [19], and in mean BP & HT [20] from population surveys in Fiji indicate continued increases in these RFs, commensurate with trends in premature adult mortality and proportional mortality from CVD [1, 21].

WHO STEPS surveys in 13 Pacific states during the first decade of this century indicate a median HT prevalence of 22 % (inter-quartile range 17–26 %) [22], compared with the 33 % in VHZ in 2012 from the present study using the same criteria. WHO STEPS surveys in the same Pacific states during this period suggest a median T2DM prevalence of 23 % (inter-quartile range 18–32 %) [22], compared with the 16 % from the present study. Recent studies of the relatively isolated Island of Wallis have documented significant increases in cardiovascular risk over 1980–2009 [23].

In Fiji and other Pacific countries such as Tonga [24, 25] and Nauru [26], NCDs are producing sufficient premature mortality to result in plateaux in LE over decades [27]. Increasing CVD proportional mortality in Fiji is already having an effect [21], as premature adult mortality has increased, and LE has not improved since 1985 in either sex [21], or ethnicity [1]. The situation is worst in i-Taukei women where recent evidence indicates LE has decreased [1].

Considerable premature adult mortality from CVD and other NCDs also contributes to relatively low and static LE trends in Indigenous minorities in developed countries, such as Australian Aboriginals [28, 29] and New Zealand Maori [30], and in some Eastern European countries (especially Russia) and central Asian states [31, 32]. Such plateaux in LE are often at lower levels and more prolonged than experienced by Australia [33–35], New Zealand, and other English-speaking during their NCD epidemic last century.

## Conclusions

The 2012 VHZ survey of NCDs and RFs resulted in findings which were quite similar to the 2011 national Fiji STEPS survey with respect to obesity, T2DM and HT, while smoking, alcohol consumption and low fruit and vegetable intake were more prevalent in VHZ. These relatively high prevalences of NCD and RFs convey risk of CVD episodes and death, and contribute to premature adult mortality and the observed plateaux in LE in Fiji since the 1980s.

The previous and present initiatives in Fiji for NCD prevention and control that require strengthening include: the SNAP (smoking, nutrition, alcohol, physical activity) framework for individual and community health promotion directed at behavioural NCD RFs; personal diabetes record books; WHO PEN protocols for RF detection and management for individuals and communities; mass media campaigns; the ‘Wellness cascade’ for health education/promotion using a life course approach; and the use of volunteer community health workers in primary health care, including NCD prevention and control [36, 37]. It is crucial to evaluate the effectiveness of these initiatives on NCD and RF prevalences and premature mortality from CVD, diabetes and other NCDs in order to continually revise and refresh approaches to prevention and control.

## Abbreviations

BMI: body mass index
BP: blood pressure
CVD: cardiovascular disease
DBP: diastolic blood pressure
FPG: fasting plasma glucose
HT: hypertension
IFCC: International Federation of Clinical Chemistry
ISH: International Society of Hypertension
LE: life expectancy
MoH: Ministry of Health
NCD: non-communicable disease
OR: odds ratio
RF: risk factor
SAS: Statistical Analysis Software
SBP: systolic blood pressure
SNAP: smoking, nutrition, alcohol, physical activity
T2DM: type 2 diabetes mellitus
VHZ: Viseisei Health Zone
WHO: World Health Organisation
